# Dog bite and injury awareness and prevention in migrant and left-behind children in China

**DOI:** 10.1038/s41598-018-34428-1

**Published:** 2018-10-29

**Authors:** Ying Chen, Yafei Tan, Shuzhen Yan, Liping Li

**Affiliations:** 1 Center for Injury Prevention Research, Shantou University Medical College, Shantou, Guangdong province China; 2 School of Public Health, Sun Yat-sen University, Guangzhou, Guangdong province China

## Abstract

Dog-incurred injury is a serious public health concern worldwide, especially for children, responsible for numerous infectious diseases, such as rabies. Our study aims to investigate the current status of dog-incurred injury and its prevention among special groups of children. A cross-sectional study was conducted among migrant children (MC) and non-MC, “left-behind” children (LBC) and non-LBC in two cities in southern China. A questionnaire was used to collect socio-demographic data and experience with dog-incurred injury, to assess knowledge of dog behaviour and rabies prevention and attitudes in risk identification and practice in risk behaviour. A total of 9,380 children aged 6–19 years old participated in this study. The self-reported prevalence of overall lifetime dog-incurred injuries in MC was 19.4% (vs. 11.2% in non-MC, χ^2^ = 175.8) and LBC was 20.6% (vs. 13.5% in non-LBC, χ^2^ = 114.1). MC were more likely to provoke a dog before the injury happened (12.7% vs 11.0%), while LBC (13.3% vs. 10.7%) and non-MC (13.1% vs. 12.2%) did not manage their wounds (all *P* < 0.001). 45.6% of victims were alone when the attack happened. MC from other provinces who live in rural areas and LBC with their mother absent had the lowest levels of knowledge among the groups. Taken together, MC and LBC are at greater risk for dog-incurred injury. They had lower knowledge of and attitudes towards prevention and more high-risk behaviour. Therefore, an appropriate public health education intervention is needed for schoolchildren regarding the prevention and management of dog-incurred injury and disease.

## Introduction

China has become the third-largest pet-owning country after the United States and Japan^1^. The rate of pet ownership in China has reached 30–40% according to surveys^2,3^. Although pets confer many benefits^4,5^, there are also adverse effects and potential risks, particularly for dog owners. The World Health Organization (WHO) report in 2013 showed that animal injury has become a major cause of morbidity and mortality for children worldwide^6^ and two-thirds of animal injuries are caused by dogs^7^. Children are the most frequent victims of dog bites because of their short stature, low awareness of danger, and curiosity for the unfamiliar^8–12^. However, children do not have the knowledge required to manage their injuries in an appropriate and timely fashion and thus, serious consequences including death may ensue. Furthermore, child victims are also more vulnerable to psychological trauma. A previous study suggested that children injured by pets are at risk for developing PTSD (post-traumatic stress disorder)^13^.

Migrant children (MC) refer to children of external workers, whose parents take them from other cities or provinces to their currently inhabited cities, which are inconsistent with their registered residence or birthplace. “Left-behind” children (LBC) refers to children whose parent or parents have left their hometown to earn a living elsewhere and cannot return home regularly, they lack long-term companionship and care from their parents, experience neglect and are vulnerable to psychological problems. MC and LBC, who are regarded as two special groups in China, constitute a population of nearly 100 million^14^ and their physical and psychological health warrants increased attention. Rapid economic development and urbanization have led to rural labourers moving into cities, which has resulted in a large number of migrant and LBC. MC and LBC are unable to obtain adequate and timely companionship and care from their parents due to personal or financial reasons, rendering them more vulnerable to psychological, social, and physical short- and long-term developmental issues^15,16^. A previous study has shown that parents working away from their hometown is a risk factor for dog-incurred injury among children^17^. It remains unknown whether migrant and LBC, two special groups, are more vulnerable to animal injuries.

Therefore, in this study, we primarily aimed to explore whether there was any difference in the characteristics of dog-incurred injuries between MC and non-MC, LBC and non-LBC and the special characteristics of the injuries according to group; secondly, to explore the differences of the knowledge, attitudes and practices towards dog-incurred injury prevention in different children groups.

## Methods

### Participants

This cross-sectional study was conducted between April and May 2015. We chose two representative cities according to their local economic development and demographic compositions, in order to ensure representativeness and unbiased sampling. The participants were enrolled from two cities located in the Guangdong province, south of China. Shenzhen, a well-developed city neighbouring Hong Kong, is one of the top four megacities in China and has a population of 10.78 million (2014), 70% of which represents migrants. Shantou, a developing city, is a relatively conservative and historical city with a population consisting mostly of original residents in which most adults travel to other cities for work, leaving many LBC. Moreover, we have conducted several studies on child injury and fostered sound networks with local schools in both cities.

A multistage sampling method was used to select the target schools. We numbered all of the schools from the two cities in different districts (divided into central urban and suburban) and randomly selected several primary, junior, and senior schools through random sampling to distribute them as evenly as possible in each district and grade. A total of 17 schools, including six primary, six junior and five senior secondary schools were selected using to this design. All of the students in grades 1–11 from 17 schools were included in this study.

A self-administered questionnaire was used to investigate child-dog interactions among the participants, which included three components: (1) Socio-demographic characteristics, including personal demographics, family factors, living conditions, pet ownership; (2) Dog-incurred injury, including prevalence of lifetime experience, past year experience, injury characteristics and treatment details; (3) Knowledge of, attitudes towards, and practice of prevention in dog-incurred injury. We performed a test-retest and the results showed that the correlation coefficient ranged from 0.786–0.851 (*p* < 0.01). Exploratory factor analysis was performed to assess the construct validity of the questionnaire and the results indicated good construct validity^18^.

In order to ensure the data quality of the questionnaire, we designed a student and parent version. Children in grades 4–11 (aged 10 years and above) completed their questionnaires in the classroom, with the assistance of trained investigators if needed. While younger students (grades 1–3) took the questionnaires home and completed them together with their parents. All completed questionnaires were checked immediately after collection for accuracy and completeness.

### Measurements

#### Definition of MC and LBC

LBC refers to children whose parent or parents have left their hometown to earn a living elsewhere and cannot return home regularly, they lack long-term companionship and care from their parents, experience neglect and are vulnerable to psychological problems. LBC were classified into three groups according to caretaker status—fathers away, mothers away, both away (leave to other guardians). MC was defined as children of external workers, whose parents take them from other cities or provinces to their currently inhabited cities, which are inconsistent with their registered residence or birthplace. They do not enjoy the same educational opportunities and social resources and are also excluded from the formal education system in rural areas, thus becoming a marginalized group. “Other cities” refers to cities other than those in which the survey was conducted (cities in Guangdong province, except Shenzhen and Shantou city); “other provinces” refers to provinces other than those in which the survey is conducted (provinces in China, except Guangdong province).

#### Socio-demographic characteristics

The data collected for this study involved socio-demographics including familial and personal variables: current resident (address, city), sex, grade, single-child family, personality, interest in animals, academic performance, parents’ educational level, parents’ occupation, average monthly income and pet ownership.

#### Prevalence and characteristics of dog-incurred injury experiences

We calculated the prevalence of dog-incurred injuries in the participants’ entire lives and defined this as “lifetime injury”. An injury was defined as “past-year injury” if it happened within the past 12 months. Injury was considered to be any non-fatal physical damage to a child’s body caused by a dog that the children could recall precisely (e.g. bites, scratches, falls caused by a dog).

#### Assessment of knowledge, attitudes, and practice (KAP)

A KAP survey is a representative study of a specific population that reflects information on what is known, believed and done in relation to a particular topic; data are collected orally by an interviewer using a structured, standardized questionnaire^19^. In this study, we explored the KAP or risk behaviour in terms of dog-incurred injury among different groups of children. The Knowledge portion was composed of 20 questions (10 items about knowledge on judging dog behaviour, 10 items about knowledge on rabies prevention). All of the participants were required to choose the most suitable answer to every question based on their own situation. The total scores ranged from 0–20. The scores were used to assess the knowledge level of children and a higher score was indicative of more knowledge.

The Attitudes portion was assessed using 17 items that described one’s awareness before danger occurred, such as “When a dog comes towards me, I can shout and scare it away” and “If I’ve received the rabies vaccine, I can play with the dog wildly”, etc. The participants were instructed to choose a description of the attitude closest to theirs (Totally agree = 1, Agree = 2, Not sure = 3, Disagree = 4, Totally disagree = 5). The total scores ranged from 17–85. Higher scores indicated greater levels of prevention awareness.

The Practice portion was used to determine behaviour in terms of contacting a dog directly or indirectly in reality. For example, “Playing with a dog or playing beside a dog”, “Feeding the dog” and “Teasing an unfamiliar dog just for fun”, etc. Five frequency levels (Never = 1, Once every 3 months = 2, Once every 3 weeks = 3, Once every week = 4, Everyday = 5) were used to describe 15 risky behaviour items. The total score ranged from 15–75. Higher scores indicated more frequently occurring risky behaviour. All of above question were set up as single or multiple choice.

### Statistical analysis

Data were analysed using the SPSS version 22.0. (SPSS Inc.: Chicago, IL, USA). Descriptive statistics were used to compare the demographic characteristics and the prevalence of dog-incurred injury in the different groups (e.g. non-MC, MC, non-LBC and LBC). The chi-square test was used to compare frequencies of the categorical variables between non-MC and MC and between non-LBC and LBC. One-way analysis of variance (ANOVA) was used to evaluate the KAP scores. Spearman’s rank correlation coefficient was used to identify whether variables relate in a monotonic function and to calculate the r-value for evaluating the correlation. A significance level of *p* < 0.05 was adopted. Due to space limitations, only values above 20% were presented in the tables.

### Ethical considerations

Written informed consent forms were obtained from all study participants or their parents prior to the investigation. This study was approved by the Ethical Committee of the Shantou University Medical College (SUMC-2015–41). All procedures were conducted in accordance with the Declaration of Helsinki.

## Results

A total of 9,788 (99.6%) students from 17 schools agreed to participate in the study and 9,380 (95.8%) cases with valid data were analysed. All participants were 6–19 years old (mean age = 12.8 years, median = 13.0 years) with similar proportions of boys and girls (50.5% vs. 49.5%). There were statistically significant differences between all of the groups (MC and non-MC, LBC and non-LBC) with the whole demographic characteristics except for “interest in animals” between LBC and non-LBC (Table 1).

**Table 1:** The personal information of the study sample stratified by migrant (n = 8318) and left behind (n = 9092) status.

Demographic characteristics N (%)	Non-MC	MC (n = 3464, 41.6%)		Non-LBC	LBC (n = 2193, 24.1%)		
		From other cities	From other provinces		Father Away	Mother Away	Both Away
	4854 (58.4)	1802 (21.7)	1662 (19.9)	6899 (75.9)	884 (9.7)	127 (1.4)	1182 (13.0)
Sex							
Boy	2278 (46.9)	938 (52.1)	919 (55.3)	3417 (49.5)	418 (47.3)	74 (58.3)	683 (57.8)
Girl	2576 (53.1)	864 (47.9)	743 (44.7)	3482 (50.5)	466 (52.7)	53 (41.7)	499 (42.2)
Age							
6~9	385 (7.9)	419 (23.2)	417 (25.1)	839 (12.2)	99 (11.2)	18 (14.2)	352 (29.8)
10~13	665 (13.8)	457 (25.4)	416 (25.0)	1263 (18.3)	176 (19.9)	27 (21.3)	286 (24.2)
14~15	1769 (36.4)	331 (18.4)	454 (27.3)	2264 (32.8)	196 (22.2)	37 (29.1)	315 (26.6)
16~19	2035 (41.9)	595 (33.0)	375 (22.6)	2533 (36.7)	413 (46.7)	45 (35.4)	229 (19.4)
City							
Shenzhen	1303 (26.8)	1586 (88.0)	1509 (90.8)	3457 (50.1)	341 (38.6)	79 (62.2)	957 (81.0)
Shantou	3551 (73.2)	216 (12.0)	153 (9.2)	3442 (49.9)	543 (61.4)	48 (37.8)	225 (19.0)
Living area							
Urban	1770 (36.5)	1110 (61.6)	561 (33.8)	3114 (45.1)	308 (34.8)	49 (38.6)	321 (27.2)
Rural	3084 (63.5)	692 (38.4)	1101 (66.2)	3785 (54.9)	576 (65.2)	78 (61.4)	861 (72.8)
Single-child family							
Yes	1473 (32.1)	171 (10.0)	379 (24.0)	1772 (27.7)	161 (19.8)	41 (36.9)	184 (16.7)
No	3118 (67.9)	1531 (90.0)	1201 (76.0)	4636 (72.3)	652 (80.2)	70 (63.1)	921 (83.3)
Interest in animal							
Dislike	466 (9.7)	164 (9.1)	115 (7.0)	609 (8.9)*	87 (9.9)*	9 (7.1)*	111 (9.5)*
Normal	2005 (41.6)	788 (44.1)	606 (36.8)	2827 (41.2)*	375 (42.7)*	54 (42.5)*	467 (39.9)*
Like	2348 (48.7)	836 (46.8)	924 (56.2)	3421 (49.9)*	417 (47.4)*	64 (50.4)*	592 (50.6)*
Academic performance							
Good	2342 (49.5)	937 (53.0)	713 (43.6)	3429 (50.7)	387 (44.6)	39 (31.2)	454 (39.0)
Average	1461 (30.9)	535 (30.3)	482 (29.4)	2049 (30.4)	310 (35.7)	43 (34.4)	307 (26.4)
Poor	928 (19.6)	296 (16.7)	442 (27.0)	1280 (18.9)	171 (19.7)	43 (34.4)	404 (34.6)

*All the group have the significance that *P* < 0.0001, except with *indicate *P* = 0.700.

### Demographic characteristics of MC and LBC

Compared to non-MC, there were more boys migrated in early age and non-single child families. Most MC from other provinces were more likely to live in rural areas and have dogs. Non-MC were less willing to have dogs and their parents had higher educational levels (Table 2). Similar to the MC, there were more boys than girls among the LBC. Compared to other age groups, both parents working away accounted for the largest proportion (29.8%) of children who were in early primary school. The likelihood of a children’s family raising a dog was: parents both away > mothers away > fathers away > non-LBC (no parent away).

**Table 2:** The family information of the study sample stratified by migrant (n = 8318) and left behind (n = 9092) status.

Demographic characteristics N (%)	Non-MC	MC (n = 3464, 41.6%)		Non-LBC	LBC (n = 2193, 24.1%)		
		From other cities	From other provinces		Father Away	Mother Away	Both Away
	4854 (58.4)	1802 (21.7)	1662 (19.9)	6899 (75.9)	884 (9.7)	127 (1.4)	1182 (13.0)
Father’s educational level							
Primary school or less	522 (11.3)	173 (9.8)	197 (12.2)	703 (10.5)	115 (13.5)	24 (19.7)	163 (14.2)
Junior high school	1607 (34.7)	803 (45.7)	649 (40.3)	2467 (37.1)	331 (38.8)	45 (36.9)	527 (45.9)
Senior high school	1376 (29.7)	591 (33.6)	517 (32.2)	2077 (31.1)	268 (31.4)	32 (26.2)	354 (30.8)
University or above	1124 (24.3)	191 (10.9)	246 (15.3)	1423 (21.3)	139 (16.3)	21 (17.2)	105 (9.1)
Mother’s educational level							
Primary school or less	1065 (23.0)	389 (22.3)	347 (21.8)	1411 (21.2)	269 (31.9)	22 (18.4)	297 (26.2)
Junior high school	1461 (31.6)	784 (45.0)	641 (40.3)	2350 (35.3)	282 (33.5)	45 (37.5)	498 (44.0)
Senior high school	1205 (26.1)	423 (24.3)	432 (27.2)	1752 (26.3)	194 (23.0)	34 (28.3)	271 (23.9)
University or above	894 (19.3)	146 (8.4)	171 (10.7)	1137 (17.2)	98 (11.6)	19 (15.8)	67 (5.9)
Average monthly income							
Low	2810 (62.6)	851 (49.8)	855 (53.6)	3686 (57.2)	474 (57.1)	80 (65.6)	712 (62.3)
Average	1142 (25.4)	590 (34.5)	526 (33.0)	1865 (28.9)	244 (29.4)	30 (24.6)	333 (29.2)
High	537 (12.0)	269 (15.7)	214 (13.4)	894 (13.9)	112 (13.5)	12 (9.8)	97 (8.5)
Parents’ marital status							
In marriage	4519 (95.0)	1709 (96.9)	1507 (95.2)	6498 (96.1)	806 (93.5)	91 (72.8)	1088 (96.2)
Divorce	156 (3.3)	44 (2.5)	64 (4.0)	181 (2.7)	46 (5.3)	27 (21.6)	32 (2.8)
One or both died	80 (1.7)	11 (0.6)	12 (0.8)	81 (1.2)	10 (1.2)	7 (5.6)	11 (1.0)
Raising pets							
Never	2005 (41.9)	896 (50.3)	825 (50.4)	3008 (44.2)	396 (45.2)	46 (36.5)	586 (50.5)
Ever but not now	1743 (36.4)	505 (28.4)	466 (28.4)	2287 (33.5)	312 (35.6)	45 (35.7)	300 (25.8)
Currently dog	320 (6.7)	147 (8.3)	201 (12.3)	512 (7.5)	57 (6.5)	15 (11.9)	155 (13.4)
Currently cat	399 (8.3)	125 (7.0)	68 (4.2)	535 (7.8)	65 (7.4)	14 (11.1)	68 (5.9)
Currently other pets	322 (6.7)	107 (6.0)	78 (4.7)	478 (7.0)	46 (5.3)	6 (4.8)	51 (4.4)

*All the group have the significance that *P* < 0.0001.

### The lifetime and past-time prevalence of MC and LBC injured by a dog

**Figure 1:**
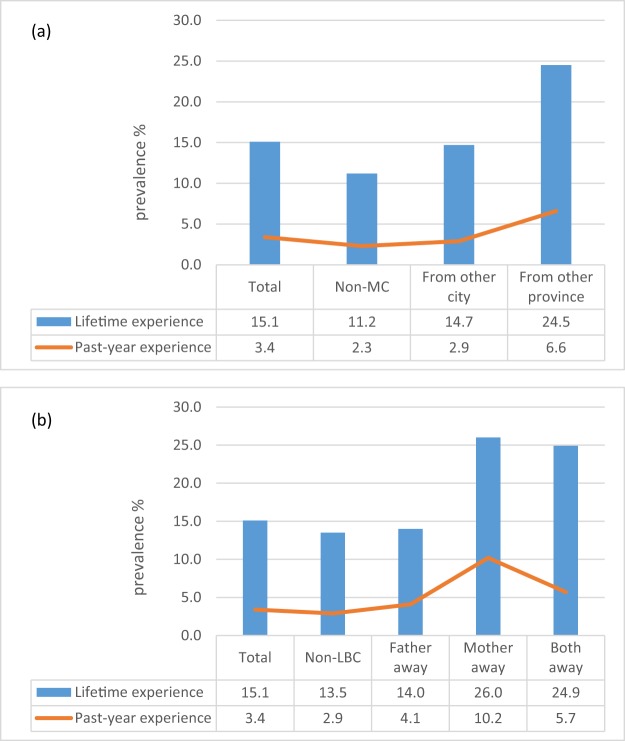
The prevalence of dog-induced injury for migrant children (**a**) and left-behind children (**b**) in lifetime and past-time among 9380 children in two southern cities of China.

Figure 1 shows the significant difference in self-reported prevalence of injury from a dog between MC and non-MC in lifetime and past-year experiences. Similarly, there were significant differences in the self-reported prevalence of injury from a dog between LBC and non-LBC in lifetime and past-year experiences. Statistically significant differences were found between all groups.

### Characteristics of dog-incurred injury in MC and LBC

Table 3 demonstrates that among the 1,413 participants injured by a dog, the leading cause of dog-incurred injury was a bite (57.5%) and 49.4% of injuries were caused by other families’ dogs. 45.6% of victims were alone when the attack happened. Compared with non-MC and non-LBC, MC and LBC had a higher proportion of causing the injury while they were eating (4.2% vs. 2.4%, *p* < 0.005) or harming the dog without intention (11.2% vs. 5.1%, *p* < 0.05). MC were more likely to provoke the dog before the injury happened (12.7%). As for the location of the occurrence, the dog-incurred injuries in MC and LBC were more likely to happen in public places, while non-MC were in side streets or the dogs’ home, and about half of the injuries damaged the skin. More MC dealt with their wounds themselves than non-MC (21.2% vs. 14.9%), but more non-MC made no efforts to manage their wounds (13.3% vs. 10.7%, *p* < 0.0001) and were less likely to go to a clinic or emergency department than MC (46.7% vs. 51.4%). Moreover, non-MC did not immediately inform their parents or guardians of being injured (66.5% vs. 75.4%, *p* = 0.002) and even left them uninformed (66.5% vs. 75.4%).

**Table 3:** Characteristics of dog-induced injury in migrant and left-behind children among two southern cities in China.

N (%)	Total	Migrant status			LBC status		
		MC	Non-MC	*p*-value	LBC	Non-LBC	*p*-value
	n = 1413	n = 673	n = 545		n = 451	n = 930	
What was kind of injury type?				0.393			0.264
Bite	812 (57.5)	392 (58.2)	318 (58.3)		249 (55.2)	542 (58.3)	
Scratch	360 (25.5)	175 (26.0)	125 (22.9)		112 (24.8)	240 (25.8)	
Fall	125 (8.8)	59 (8.8)	53 (9.7)		44 (9.8)	79 (8.5)	
Bite and scratch	116 (8.2)	47 (7.0)	49 (9.0)		46 (10.2)	69 (7.4)	
Where was the dog from?				0.343			0.398
Own family	425 (30.3)	188 (28.2)	168 (31.0)		131 (29.3)	288 (31.2)	
Other families	693 (49.4)	338 (50.7)	284 (51.9)		220 (49.2)	456 (49.4)	
Stray	131 (9.3)	65 (9.7)	43 (7.9)		39 (8.7)	89 (9.6)	
Unknown	154 (11.0)	76 (11.4)	50 (9.2)		57 (12.8)	91 (9.8)	
What were you doing when injury happening?				0.002			0.017
Walking pass by	325 (24.1)	152 (23.9)	132 (25.0)		110 (25.8)	208 (23.4)	
Running pass by	171 (12.7)	76 (11.9)	78 (14.8)		48 (11.2)	118 (13.3)	
Playing with dog	197 (14.6)	85 (13.4)	79 (15.0)		57 (13.3)	137 (15.4)	
Provoking dog	160 (11.9)	81 (12.7)	58 (11.0)		49 (11.5)	107 (12.1)	
Attacking dog	57 (4.2)	21 (3.3)	25 (4.7)		17 (4.0)	40 (4.5)	
Feeding, washing, training dog	79 (5.9)	40 (6.3)	29 (5.5)		18 (4.2)	59 (6.7)	
Eating something	40 (3.0)	23 (3.6)	10 (1.9)		18 (4.2)	21 (2.4)	
Playing	140 (10.4)	65 (10.2)	55 (10.4)		49 (11.5)	87 (9.8)	
Harming dog without intention	113 (8.4)	71 (11.2)	27 (5.1)		48 (11.2)	61 (6.9)	
Others	64 (4.8)	22 (3.5)	34 (6.5)		13 (3.0)	49 (5.5)	
Who companying with you when injury happened?				0.033			0.839
Alone	645 (46.5)	293 (44.5)	269 (50.1)		195 (44.5)	437 (47.7)	
Parent	255 (18.4)	136 (20.6)	84 (15.6)		85 (19.5)	167 (18.2)	
Grandparent	150 (10.8)	79 (12.0)	46 (8.6)		44 (10.1)	99 (10.8)	
Playmate	241 (17.4)	103 (15.6)	104 (19.4)		82 (18.8)	152 (16.6)	
Others	95 (6.9)	48 (7.3)	34 (6.3)		31 (7.1)	62 (6.7)	
What time is injury happening?				0.003			0.618
7:00–15:00	531 (40.8)	275 (44.4)	194 (38.3)		173 (41.8)	348 (40.6)	
15:00–21:00	650 (50.0)	283 (45.7)	280 (55.2)		201 (48.6)	438 (51.0)	
21:00 - next day 7:00	119 (9.2)	61 (9.9)	33 (6.5)		40 (9.7)	72 (8.4)	
Where is injury happening?				0.008			0.013
Own home	393 (28.5)	182 (27.7)	148 (27.8)		126 (28.8)	262 (28.8)	
Side of street	481 (34.9)	227 (34.6)	198 (37.2)		145 (33.1)	319 (35.1)	
Public place	156 (11.3)	88 (13.4)	37 (7.0)		65 (14.8)	88 (9.7)	
Dog’s home	248 (18.0)	117 (17.8)	108 (20.3)		64 (14.6)	178 (19.6)	
Other places	101 (7.3)	43 (6.5)	41 (7.7)		38 (8.7)	62 (6.8)	
What about the wound?				0.309			0.315
Skin unbroken	716 (52.7)	336 (51.8)	285 (54.7)		227 (52.2)	474 (53.1)	
Leaving a mark without bleeding	380 (28.0)	187 (28.8)	136 (26.1)		116 (26.7)	253 (28.4)	
Skin broke with a little blood	117 (8.6)	62 (9.6)	39 (7.5)		35 (8.0)	78 (8.7)	
Lacerated wound with a lot of blood	146 (10.7)	64 (9.9)	61 (11.7)		57 (13.1)	87 (9.8)	
How long the guardians know after you injured by dog?				0.002			0.736
Immediately	970 (70.8)	493 (75.4)	350 (66.5)		319 (72.7)	633 (70.3)	
Within 12 hours	140 (10.2)	61 (9.3)	61 (11.6)		38 (8.7)	98 (10.9)	
After 12–24 hours	52 (3.8)	22 (3.4)	21 (4.0)		18 (4.1)	32 (3.6)	
After 24 hours	41 (3.0)	19 (2.9)	11 (2.1)		13 (3.0)	27 (3.0)	
Unknown	167 (12.2)	59 (9.0)	83 (15.8)		51 (11.6)	110 (12.2)	
What about the wound management?				<0.0001			0.619
No management	168 (12.4)	69 (10.7)	69 (13.3)		57 (13.1)	108 (12.2)	
Cared by myself	253 (18.7)	137 (21.2)	77 (14.9)		87 (20.0)	157 (17.7)	
Managed with other adults’ help	281 (20.8)	108 (16.7)	130 (25.1)		90 (20.7)	184 (20.7)	
Cared by clinic	651 (48.1)	332 (51.4)	242 (46.7)		200 (46.1)	439 (49.4)	

### KAPs stratified by MC and LBC

In terms of the children’s education level, the participants stratified into two groups according to living area (urban vs. rural) (Table 4). There were significant differences between all of the groups (*p* < 0.05). In terms of knowledge of dog behaviour and rabies prevention, children living in rural areas had lower scores than those living in urban areas (*t* = 10.942, *p* < 0.0001). In particular, MC who lived in rural areas from other provinces and LBC with mothers away had the lowest scores on knowledge among all of the groups. The questions with lower scores were: a. A dog suddenly wants to attack you, the reason for the attack may be? b. Before playing with dogs, what is the appropriate behaviour/should be noted? c. How long is it necessary to have received the rabies vaccine after being bitten by an animal? d. After being bitten by an animal, which of the following measures to prevent rabies is ineffective?

**Table 4:** Knowledge, attitude and practice of two cities’ children on dog-induced injury stratified by urban and rural areas.

		Urban area (n = 4967)				Rural area (n = 4413)			
		Number	Knowledge	Attitude	Practice	Number	Knowledge	Attitude	Practice
**Non-MC**		1770	10.6 ± 3.5	68.9 ± 9.3	26.4 ± 11.7	3084	9.8 ± 3.4	66.9 ± 7.6	26.4 ± 10.1
**MC**	Other cities	1110	9.9 ± 3.3	69.9 ± 8.2	23.0 ± 9.8	692	9.4 ± 3.5	68.0 ± 8.8	27.8 ± 12.3
	Other provinces	561	10.3 ± 3.5	68.7 ± 9.2	25.5 ± 11.9	1101	8.9 ± 3.4	67.5 ± 8.3	27.5 ± 11.8
	*F*		12.317	5.294	30.733		29.056	6.599	6.420
	*p*-value		<0.0001	0.005	<0.0001		<0.0001	0.001	0.002
**Non-LBC**		3114	10.4 ± 3.5	69.4 ± 9.1	25.3 ± 11.4	3785	9.6 ± 3.4	67.3 ± 7.9	27.0 ± 10.8
**LBC**	Father away	308	9.8 ± 3.4	67.7 ± 8.4	26.4 ± 12.1	576	9.2 ± 3.5	66.3 ± 7.9	26.1 ± 10.9
	Mother away	49	9.6 ± 4.0	64.4 ± 10.0	27.1 ± 10.5	78	8.0 ± 4.0	63.7 ± 9.6	29.7 ± 11.2
	Both away	321	9.6 ± 3.8	68.0 ± 8.9	22.9 ± 10.1	861	8.9 ± 3.5	67.0 ± 8.5	27.3 ± 11.9
	*F*		8.118	9.520	5.700		14.483	6.985	2.774
	*p*-value		<0.0001	<0.0001	0.001		<0.0001	<0.0001	0.040

For the attitudes towards dog behaviours and rabies prevention, the participants who registered household in other cities (Mean (M) = 69.2, Standard deviation (*SD)* = 8.4) were significantly different from those who registered in other provinces (*M* = 67.9, *SD* = 8.6) or from non-migrant families (*M* = 67.6, *SD* = 8.4). The children without parents away held the highest scores among all groups. Similar to the knowledge portion, LBC with mothers away for work had the most uninformed prevention attitudes. The questions with lower scores were: a. Compared to other dogs, I think that my dog is less likely to bite others; b. Smaller dogs are less likely to cause me harm; c. I believe that domestic dogs in the city should undergo obedience training; d. I think that every domestic dog owner should be vaccinated against rabies; e. I think that dog owners should use leashes when they are walking outdoors.

A comparison of current residence revealed that all children living in rural areas were more frequently exposed to dogs than those living in urban areas (*t* = −6.907, *p* < 0.0001). According to the correlation analysis, the children with higher knowledge scores tended to hold better attitudes towards preventing dog-incurred injury (*r* = 0.35, *p* < 0.0001) but there was no significant association between knowledge and risky behaviour among children (*r* = 0.014, *p* = 0.187). As expected, attitude scores negatively correlated with behaviour scores (*r* = −0.203, *p* < 0.0001). The most common dangerous behaviours were: a. Running around a cat or dog; b. Riding past a cat or dog; c. Playing with a cat or dog, or playing near a cat or dog.

## Discussion

This is the first cross-sectional study based on specific populations to focus on the current status of dog-incurred injury and assess the knowledge, attitudes and practices of MC and non-MC, and LBC and non-LBC. We found that both MC and LBC experienced significantly higher rates of dog-incurred injury compared to non-MC and non-LBC. MC from other provinces and those whose mothers were away were at high-risk for dog-incurred injuries. The scores on knowledge and attitudes towards dog-incurred injury and rabies prevention among MC and LBC were lower than non-MC and non-LBC but they had higher risk behaviour scores.

The causes of dog-incurred injury were different in the two groups. MC were more likely to provoke dogs than LBC. Several surveys showed that MC were more likely to suffer from behaviour problems, such as physical inactivity, internet addiction, smoking tobacco, suicide ideation, and being overweight^15,20,21^, which means that they might be more likely to have aggressive behaviour. Our results are consistent with these findings, for example, that MC might be more willing to provoke dogs. In terms of risk behaviours, LBC were more likely to play with dogs than other groups, probably because they might need companionship and consolation of emotional needs through keeping pets, which resulted in more chances of suffering from a dog-incurred injury. LBC have been found to be more vulnerable to psychological problems such as loneliness, depression, anxiety, and introversion^18,22^, which might be mitigated by pet ownership.

Many MC and LBC reported that the reasons they were injured by dogs were that they hurt dogs unintentionally at first, which means that they might be curious about dogs but do not grasp their behaviours accurately^23^. Furthermore, dog-incurred injury among MC and LBC occurred mainly in public places, while the places of injury among non-MC and non-LBC were in the dog’s home. Regarding whether guardians knew their child was injured by dogs in a timely fashion, a high proportion of non-MC were unwilling to tell their guardians when they were injured by dogs. Additionally, it was noteworthy that a high percentage of both MC and LBC treated their wounds by themselves after they were injured. Our findings suggested that MC and LBC had poorer performances on the KAPs on dog-incurred injury than non-MC and non-LBC. This knowledge on the status quo of children beliefs and practices on dog-incurred injury, especially for MC and LBC, will greatly inform future intervention measures towards reducing the occurrence of dog-incurred injury in children effectively.

Mothers play an important role in families by caring for and educating children^24^. Our study suggested that mothers also help to prevent dog-incurred injuries. Our results showed that children without parental care and custody, especially for mothers, are more likely to interact and play with dogs and other pet animals and had a high risk for dog-incurred injury. Moreover, LBC and MC, due to a lack of parental presence, fulfilled companionship and emotion needs with pets^25,26^. However, this phenomenon contributed to the high risk of dog-incurred injury among children, especially for those who failed to receive daily care and custody from their parents^27,28^. Moreover, these children, more often than not, tended to have lower KAPs of dog-incurred injury. Therefore, it is of profound importance to emphasize the role of parents in the care of their children in terms of preventing injury.

Our study also revealed a lack of sufficient KAPs in terms of rabies prevention among children, and knowledge on rabies prevention needs further popularization in children, especially for LBC and MC. The knowledge and behaviour of primary school children from rural areas were worse than those from urban areas. The knowledge about prevention of rabies and timely treatment of dog-incurred injury still remains largely insufficient, and further effective measurements are required to prevent rabies infections.

Several limitations must be noted. One limitation of this study is that the data were self-reported. Due to anonymity in data collection, it is possible that children, especially younger ones who completed the questionnaire with their parents’ help, feared that their parents would blame or punish them for their behaviour and thus did not truly report their risky interactions with dogs or injury experience. Recall bias is another limitation. Because the data were self-reported by children themselves, the results might be swayed by recall bias, especially for the time of the dog bite, how long guardians knew after the dog bite, medical attention sought after and actual practice with dogs. This might have biased or even undermined the authenticity of the data, though our investigators emphasized that the questionnaires must be answered truthfully. Another limitation is that we did not divide all participants into MC or LBC because of their complex backgrounds and different past experiences. Some of the children were both MC and LBC, so future research studies should include a greater variety of categories that more accurately represent the different situations of children. Despite these limitations, this study was the first systematic analysis of the prevalence and KAPs of Chinese children for dog-incurred injuries, which indicated that MC and LBC were the high-risk groups and need more attention.
